# Revealing the complete mtDNA genome sequence of Cemani chicken (*Gallus gallus*) by using Nanopore sequencing analysis

**DOI:** 10.5713/ab.23.0513

**Published:** 2024-06-25

**Authors:** Sutopo Sutopo, Dela Ayu Lestari, Asep Setiaji, Sri Rachma Aprilita Bugiwati, Muhammad Ihsan Andi Dagong, Nena Hilmia, Dani Garnida, Indrawati Yudha Asmara, Edy Kurnianto

**Affiliations:** 1 Department of Animal Science, Faculty of Animal and Agricultural Sciences, Universitas Diponegoro, Semarang, 50275, Indonesia; 2 Department of Animal Production, Faculty of Animal Science, Universitas Hasanuddin, Makassar, 90245, Indonesia; 3 Department of Animal Production, Faculty of Animal Husbandry, Universitas Padjajaran, Bandung, 45363, Indonesia

**Keywords:** Amino Acid, Cemani, Mitogenome, Mutation, Nucleotide, Phylogenetic

## Abstract

**Objective:**

This study aimed to identify, discover and explore the characteristics of the mtDNA genomes of Cemani chicken (*Gallus gallus*).

**Methods:**

This study used gDNA of Cemani chicken isolated from liver tissue. mtDNA sequencing was performed using WGS mtDNA analysis with nanopore technology by Oxford Nanopore Technologies GridION. Bioinformatics and data analysis were then performed.

**Results:**

This study showed that the length of the mtDNA genome is 16,789 bp, consisting of two ribosomal RNA (12S rRNA, 16S rRNA), 22 transfer RNA genes (*trnR*, *trnG*, *trnK*, *trnD*, *trnS*, *trnY*, *trnC, trnN, trnA, trnW, trnM, trnQ, trnl, trnL, trnV, trnF, trnP, trnT, trnE, trnL, trnS, trnH*), 13 protein-coding genes (PCGs) (*ND4l, ND3, COX3, ATP6, ATP8, COX2, COX1, ND2, ND1, CYTB, ND6, ND5, ND4*), and a noncoding control region (Dloop). Furthermore, analysis showed there were polymorphic sites and amino acid alterations when mtDNA Cemani chicken was aligned with references from GenBank.

**Conclusion:**

Site (988T>*) in *Dloop* genes and (328A>G) in *ND3* genes which alter glycine to stop codon, were specific markers found only in Cemani chicken.

## INTRODUCTION

The diversity of local chicken species in Indonesia is a wealth of Livestock Genetic Resources which has the potential to be used and developed as a source of germplasm for both conservation and utilization. This has an important and strategic meaning in the effort to encourage food security and prevent a decline in the potential of local chickens. Local chickens are known as chickens that come from the domestication of the jungle fowl (*Gallus gallus*) and are grouped into several types, including types of broiler, laying, dual-purpose and ornamental (fancy) types. According to Nataamijaya [1], there are 31 types of local Indonesian chickens that have been identified, one of which is the Cemani chicken.

Cemani chickens (Cemani in Javanese means “black”) are commonly found in the Temanggung area, Central Java, and are kept as ornamental chickens or raised to produce eggs which will be hatched. The Cemani chicken, also known as the black Kedu chicken, has specific characteristics marked by the entire color of its feather which is black. Every part of its body, from the skin and flesh to the bones, beak, cloaca, comb, face and legs, is black. The black body of the Cemani chicken is the result of a genetic mutation involving duplication in an area of the genome with 5 genes, resulting in fibromelanosis or hyperpigmentation [2,3]. One of the genes within the duplication area is endothelin 3 (EDN3) which plays a role in the formation of melanocyte-producing proteins. This causes the overexpression of melanocyte-forming proteins while the Cemani chicken is still an embryo [4]. This chicken commands a relatively high price, because apart from its exotic physical appearance, it is often used by the local rural community as a tribute at ancient religious ceremonies, especially the Cemani rooster with its black tongue.

So far, several researchers have carried out genetic characterization of local chickens, including the Cemani chicken, both qualitatively, quantitatively and molecularly. Particularly for genetic characterization using molecular techniques, it has been limited to mitochondrial DNA segments such as Dloop [5,6], or microsatellite [7]. Complete genetic information data regarding the mtDNA of Cemani chicken is not yet available. Moreover, research on genetic characterization of complete mtDNA (whole genome mtDNA) needs to be carried out to complement the results of previous studies and to enrich information regarding the existence of the Cemani chicken as one of Indonesia’s local chickens. Therefore, this study aimed to identify, discover and explore the characteristics of the mtDNA genomes of Cemani chicken (*Gallus gallus*).

## MATERIALS AND METHODS

### Ethical approval

The experimental procedures were approved by the Animal Research Ethics Committee of the Faculty of Animal and Agricultural Sciences, Universitas Diponegoro (No. 59–01/A-01/KEP-FPP).

### Tissue collection and DNA extraction

**Figure 1:**
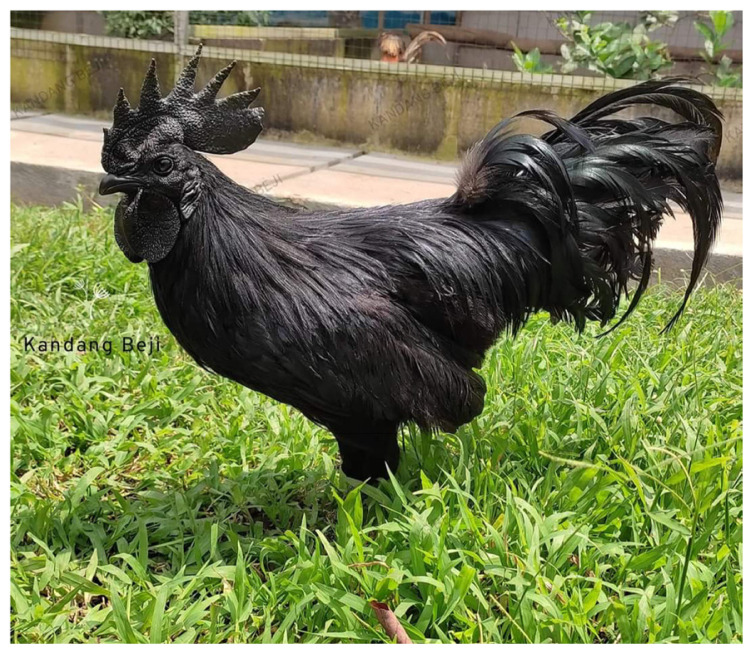
Cemani chicken (*Gallus gallus*).

This study used gDNA of Cemani chicken (Figure 1) isolated from liver tissue as it is known that cells requiring large amounts of energy tend to have a higher abundance of mtDNA. A Cemani chicken was slaughtered and dissected to collect the liver tissue. Tissue samples (10 g) were stored in Falcon tubes containing ethanol. These tissue samples were used to obtain gDNA, which was extracted according to the manufacturer’s standard protocol using the gSYNC DNA Extraction Kit (Geneaid, New Taipei, Taiwan). The collected gDNA was then selected based on quality and quantity, and genomic mtDNA enrichment was conducted using the REPLI-g Mitochondrial DNA Kit (Qiagen, Hilden, Germany). The library preparation process used the enhanced mtDNA.

### mtDNA sequencing and bioinformatic analysis

**Figure 2:**
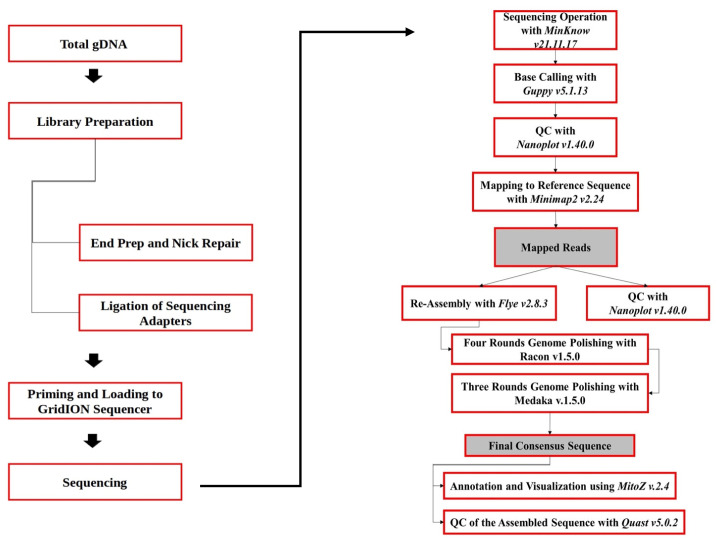
mtDNA sequencing and bioinformatic analysis procedure.

mtDNA sequencing was performed using WGS mtDNA analysis with nanopore technology [8,9] by Oxford Nanopore Technologies GridION facilitated by Genetika Science (Tangerang, Indonesia). Bioinformatics analysis was then performed. The workflow procedure for WGS, mtDNA, and bioinformatic analysis is shown in Figure 2. The MinKNOW (v21.11.17) program was used to run the sequencing output from the Oxford Nanopore Technologies GridION sequencing. Guppy (v5.1.13) performed base calling in high-accuracy mode [10]. NanoPlot (v1.40.0) was used to visualize read quality [11]. Using minimap2 (v2.24), all readings were mapped to the mitochondrial reference sequence from GenBank [12]. Flye (v2.8.3) was used to perform the assembly using the filtered mapped reads [13]. A Nanoplot (v1.40.0) was used to assess the quality of mapped and filtered reads. Racon (v1.5.0) was used to polish the constructed sequence four times and Medaka (v1.5.0) was used three times [14] (https://github.com/nanoporetech/medaka). MitoZ (v2.4) was used to annotate and visualize the final sequences [15]. Quast (v5.0.2) assessed the quality of constructed sequences [16].

### Data analysis

Data analysis was conducted using MEGA11 software [17]. Complete mtDNA genome sequences of Cemani chicken were aligned to identify the genetic characteristics, including gene sequence, position, size, amino acid length, amino acid alteration, and nucleotide composition. mtDNA genome sequences of Cemani chicken were also aligned with another 35 *Gallus gallus* complete mtDNA genome sequences obtained from GenBank as reference (Table 1) to identify mutation, diversity and visualize the genetic relationship through a phylogenetic tree constructed based on the maximum likelihood method [18].

## RESULTS AND DISCUSSION

**Figure 3:**
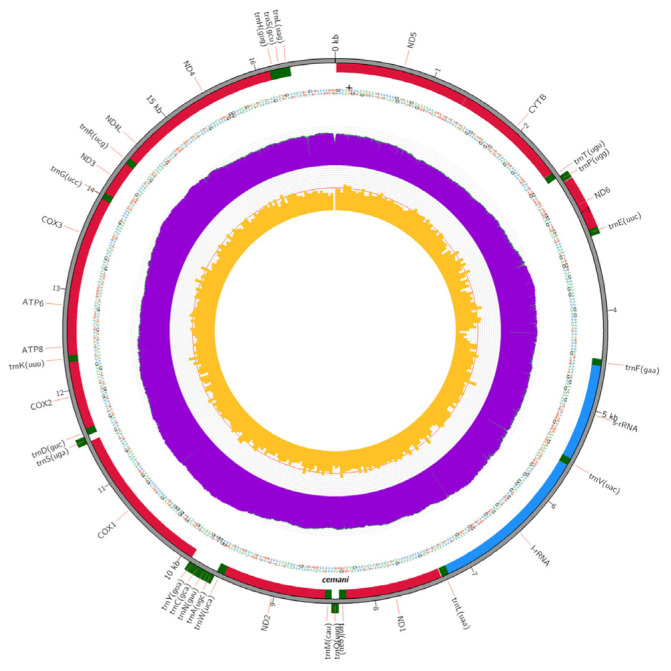
Complete mtDNA of Cemani chicken (*Gallus gallus*).

The results of the analysis showed that the length of the mtDNA genome of Cemani chicken was 16,789 bp, which consisted of 2 ribosomal RNA (l-rRNA, s-rRNA); 22 transfer RNA genes (*trnF, trnV, trnL, trnl, trnQ, trnM, trnW, trnA, trnN, trnC, trnY, trnS, trnD, trnK, trnG, trnR, trnH, trnS, trnL, trnT, trnP, trnE*); 13 protein coding genes (*ND1, ND2, COX1, COX2, ATP8, ATP6, COX3, ND3, ND4L, ND4, ND5, ND6, CYTB*) and control region (*Dloop*) (Figure 3). The results of reading the mtDNA sequences of Cemani chicken are shown in Table 2. As many as 12 of the 13 protein-coding genes of Cemani chicken begin with the ATG start codon, while the rest start with the GTG (*COX1*) start codon. The stop codons in the Cemani chicken protein coding gene were dominated by TAA (*ND1, COX2, ATP8, ATP6, ND3, ND4L, ND5, CYTB, ND6*), while the other stop codons were TAG (*ND2*); AGG (*COX1*); CTT (*COX3*); and TAT (*ND4*).

The positions of the *trnQ, trnM, trnA, trnN, trnC, trnY, trnS, trnP, trnE*, and *ND6* genes are in the light strand, while the rest are in the heavy strand. The *I-rRNA* gene is known to be the longest gene in mtDNA, which has a size of 1,620 bp. On the other hand, the shortest gene in mtDNA is the *trnC* gene, which is 66 bp long. The longest amino acid translation is owned by the *COX1* gene, which is 517 amino acids length, and the shortest amino acid translation is owned by the *ATP8* gene, which is 64 amino acids length. Overall, the DNA strand in Cemani chicken mtDNA is dominated by adenine (A) and thymine (T) bases. The most abundant composition of Adenine (A) was found in the *trnD* gene at 37.7%, and the least was found in the *ND6* gene at 10.3%. The most abundant Thymine (T) base composition was found in the *ND6* gene by 41.4%, and the least was found in the *trnF* gene. The *ATP8* gene was identified as the gene that had the least amount of Guanine (G) base composition, namely 4.8%, whereas the *ND6* gene was identified as the gene that had the most Guanine (G) base composition, namely 41.4%. Meanwhile, the most abundant composition of Cytosine (C) was found in the *ATP6* gene, namely 38.6%, and the least found in the *ND6* gene, namely 9.6%.

The overall nucleotide composition of Cemani chicken mtDNA was 23.7% for T; 32.5% for C; 30.3% for A; and 13.5% for G, which was quite similar with references as well as their mtDNA length (Table 3). The result of alignment between Cemani chicken in this study and references mtDNA nucleotide sequence from GenBank showed polymorphism in most of the genes (Table 4). Dloop gene had the highest polymorphic sites (3.65%), followed by *ND6* gene (1.91%); *ND4* gene (1.52%); *ND3* gene (1.42%); *CYTB* gene (1.31%); *ATP6* gene (1.17%); *COX1* gene (1.09%); *ND1* gene (0.92%); *COX3* gene (0.89%); *COX2* gene (0.88%); *ND2* gene (0.67%); *ND5* gene (0.44%), while *ND4L* genes had the lowest polymorphic sites (0.33%). Otherwise, *ATP8* gene showed monomorphism that indicated 100% conservation. According to the parsimony form, *ND4L* gene had the highest site (100%), due to only one mutation point. On the other hand, *ND2* genes had 71.4% parsimony form sites, while *ND4* gene had 28.57% parsimony form sites. Conversely, there was a negative correlation as in the singleton form. It was shown that *ND4* gene had the highest sites (71.42%), while *ND2* gene had the lowest sites (28.6%). This study also found indel mutation that located in the *Dloop* gene, specifically in site (859indel/C) in parsimony form and in site (860indel/C) in singleton form. Those two indel mutations were thought to have occurred due to the presence of repeat sequence of C as much as 7 to 9 times [30]. Among all polymorphic sites that found based on alignment with all references, site (988T>indel) in *Dloop* genes and site (328A>G) in *ND3* gene were specific markers that were only found in Cemani chicken and had never been reported by previous researchers. Moreover, site (988T>indel) in *Dloop* gene showed there was insertion in “T” form only in Cemani chicken, while site (328A>G) in *ND3* gene showed only Cemani chicken that had “A” in that site, while references showed “G”.

The result of alignment between Cemani chicken in this study and references mtDNA in amino acid sequence from GenBank showed there were amino acid alterations in most of the protein coding genes (PCGs) (Table 5). *ND6* gene experienced many alterations in amino acid (4.62%), followed by *ND3* gene (4.27%); *ND4* gene (1.96%); *COX1* gene (1.35%); *CYTB* gene (0.79%); *COX3* gene (0.76%); *ND5* gene (0.67%); *ND1* gene (0.61%); *COX2* and *ATP6* genes (0.44%) as the lowest. Most of the amino acid alterations in this study were due to mutations in the 1st and 2nd bases in the triplet codon unit, as stated by Nei and Kumar [41]. At the same time, amino acid alteration was not found in *ND4L* and *ATP8* genes. Those facts were due to mutation in *ND4L* gene being in the 3rd bases in the triplet codon unit leading to a synonymous form of amino acid, so that it wasn’t causing amino acid alteration, while in *ATP8* gene it was due to its gene being monomorphic. Furthermore, the notable amino acid alteration was found in *ND3* gene, specifically in codon (110AGG> GGG) which showed glycine in all references but which showed as a stop codon only in Cemani chicken.

**Figure 4:**
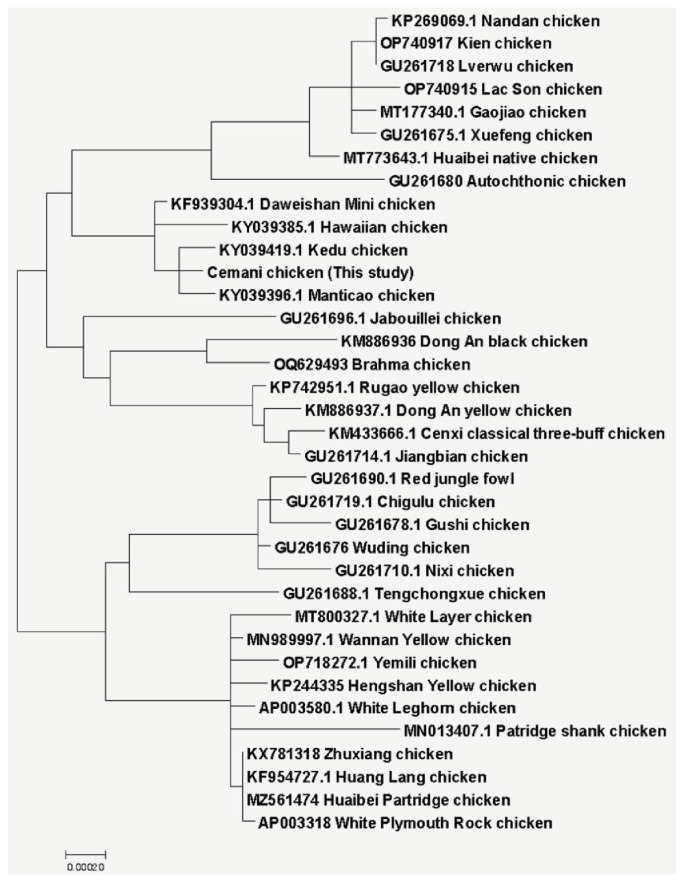
Molecular phylogenetic analysis using complete mtDNA sequence of (*Gallus gallus*) by maximum likelihood method. Cemani chicken in this study together with Kedu chicken from Indonesia and Manticao chicken from Philipine were in the same sub-sub cluster. They were also have a slightly close genetic relationship with Daweishan Mini chicken from India and Hawaiian chicken.

**Figure 5:**
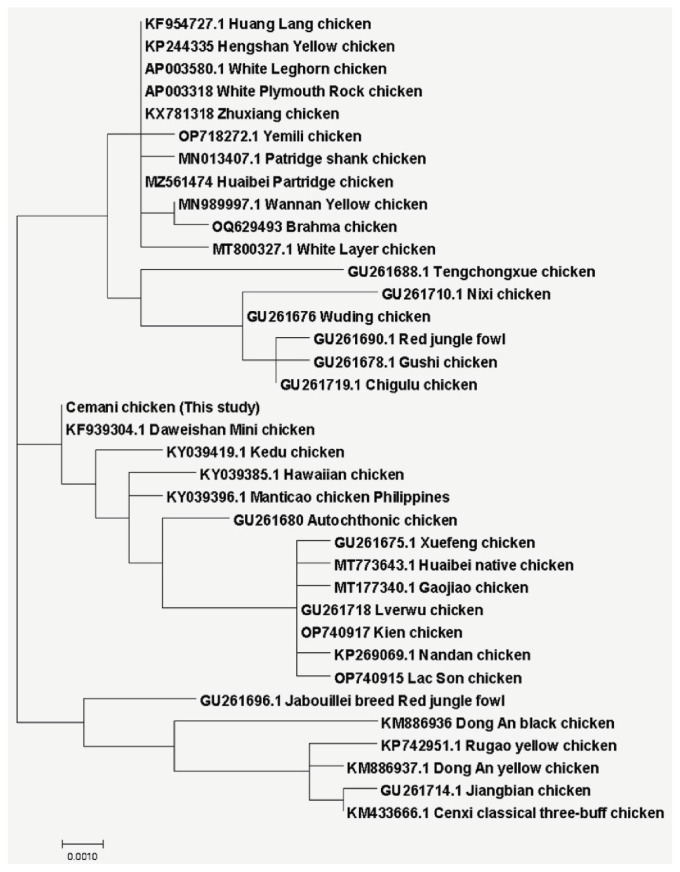
Molecular Phylogenetic analysis using *Dloop* sequence of (*Gallus gallus*) by maximum likelihood method. Cemani chicken in this study was in the same cluster as Daweishan Mini chicken, while Kedu chicken and Manticao chicken formed another sub-cluster together with Hawaiian and Autochthonic chicken.

Based on phylogenetic analysis, Cemani chicken had a very close relationship with Kedu chicken from Indonesia and Manticao chicken from the Philippines, which were still in the Southeast Asia region (Figure 4). Moreover, Daweishan Mini chicken from India and Hawaiian chicken were also known to have a slightly close relationship with Cemani chicken, because they were still in the same branch of phylogenetic tree based on Tamura and Nei [18] methods. In addition, Figure 5 showed the phylogenetic tree based on the D-loop sequence, which indicated three main clusters differently. It showed that Cemani chicken was in the same cluster as Daweishan Mini chicken, while Kedu chicken and Manticao chicken formed another sub-cluster.

## CONCLUSION

The results of the analysis showed that the length of the mtDNA genome of Cemani chicken was 16,789 bp. Site (988T>*) in *Dloop* gene and (328A>G) in *ND3* gene, which alter the stop codon to glycine, were specific markers that were only found in Cemani chicken.

## Figures and Tables

**Table 1:** *Gallus gallus* complete mtDNA sequence as references from Genbank

No	Genbank ID	Breed
1	GU261688.1	Tengchongxue chicken
2	GU261690.1	Red Jungle Fowl
3	KY039419	Kedu chicken
4	KF939304	Daweishan Mini chicken
5	OP718272	Yemili chicken
6	KX781318	Zhuxiang chicken
7	MT800327	White Layer chicken
8	AP003580.1	White Leghorn
9	GU261676	Wuding chicken
10	GU261710	Nixi chicken
11	MN013407	Partridge shank chicken
12	GU261680	Autochthonic chicken
13	GU261718	Lv’erwu chicken
14	GU261696	Jabouillei chicken
15	KF954727	Huang Lang chicken
16	KM433666	Cenxi classical three-buff chicken
17	KM886937	Dong An Yellow chicken
18	KP269069	Nandan chicken
19	KP742951	Rugao Yellow chicken
20	MT773643.1	Huaibei Native chicken
21	MT177340.1	Gaojiao chicken
22	MN989997	Wannan Yellow chicken
23	MZ561474	Huaibei Partridge chicken
24	OP740915	Lac Son chicken
25	OP740917	Kien chicken
26	AP003318	White Plymouth Rock chicken
27	GU261675	Xuefeng chicken
28	GU261678	Gushi chicken
29	KM886936	Dong An Black chicken
30	KP244335	Hengshan Yellow chicken
31	GU261719.1	Chigulu chicken
32	GU261714.1	Jiangbian chicken
33	OQ629493	Brahma chicken
34	KY039396.1	Manticao chicken
35	KY039385.1	Hawaiian chicken

**Table 2:** Complete mtDNA genome sequence profile of Cemani chicken (*Gallus gallus*)

Genes^1)^	Position		Size (bp)	Amino acid			Strand^2)^	Nucleotide composition (%)			
		
	Start	End		Length	Start codon	Stop codon		A	T	G	C
*Dloop*	1	1,232	1,232					26.5	33.4	13.5	26.6
*trnF (gaa)*	1,233	1,302	70				+	30.0	18.6	21.4	30.0
*s-rRNA*	1,302	2,278	977				+	32.3	20.4	18.2	29.1
*trnV(uac)*	2,278	2,350	73				+	34.2	20.5	17.8	27.4
*l-rRNA*	2,355	3,974	1,620				+	33.6	20.3	18.0	28.0
*trnL (uaa)*	3,976	4,049	74				+	25.7	23.0	23.0	28.4
*ND1*	4,059	5,033	975	324	ATG	TAA	+	27.1	25.2	12.8	34.9
*trnI (gau)*	5,034	5,105	72				+	37.5	20.8	19.4	22.2
*trnQ(uug)*	5,112	5,182	71				−	28.2	38.0	22.5	11.3
*trnM(cau)*	5,181	5,249	69				−	29.0	23.2	17.4	30.4
*ND2*	5,250	6,290	1,041	346	ATG	TAG	+	32.7	22.9	8.7	35.7
*trnW(uca)*	6,289	6,364	76				+	36.8	27.6	14.5	21.1
*trnA(ugc)*	6,371	6,439	69				−	24.6	34.8	24.6	15.9
*trnN(guu)*	6,443	6,515	73				−	27.4	30.1	26.0	16.4
*trnC(gca)*	6,517	6,582	66				−	25.8	30.3	27.3	16.7
*trnY(gua)*	5,683	6,652	70				−	20.0	37.1	25.7	17.1
*COX1*	6,654	8,205	1,552	517	GTG	AGG	+	27.4	25.6	15.9	31.1
*trnS(uga)*	8,197	8,271	75				−	25.3	32.0	26.7	16.0
*trnD(guc)*	8,275	8,343	69				+	37.7	21.7	15.9	24.9
*COX2*	8,344	9,027	684	227	ATG	TAA	+	29.4	23.0	14.5	33.2
*trnK(uuu)*	9,029	9,096	68				+	30.9	20.6	20.6	27.9
*ATP8*	9,098	9,262	165	54	ATG	TAA	+	34.5	24.2	4.8	36.4
*ATP6*	9,252	9,936	684	227	ATG	TAA	+	28.8	22.5	10.1	38.6
*COX3*	9,936	10,719	784	261	ATG	CTT	+	27.8	22.7	15.9	33.5
*trnG(uuc)*	10,720	10,788	69				+	33.3	29.0	13.0	24.6
*ND3*	10,789	11,140	352	117	ATG	TAA	+	28.1	26.7	12.8	32.4
*trnR(ucg)*	11,142	11,209	68				+	33.8	27.9	14.7	23.5
*ND4L*	11,210	11,506	297	98	ATG	TAA	+	27.9	24.9	12.5	34.7
*ND4*	11,500	12,877	1,378	459	ATG	TAT	+	29.9	23.6	10.2	36.3
*trnH(gug)*	12,878	12,946	69				+	33.3	30.4	14.5	21.7
*trnS(gcu)*	12,947	13,013	67				+	26.9	20.9	22.4	29.9
*trnL(uag)*	13,014	13,084	71				+	35.2	26.8	19.7	18.3
*ND5*	13,550	14,902	1,353	450	ATG	TAA	+	30.7	22.2	11.2	35.8
*CYTB*	14,907	16,049	1,143	380	ATG	TAA	+	27.5	24.0	12.1	36.5
*trnT(ugu)*	16,053	16,121	69				+	37.7	29.0	13.0	20.3
*trnP(ugg)*	16,122	16,191	70				−	24.3	32.9	28.6	14.3
*ND6*	16,198	16,719	522	173	ATG	TAA	−	10.3	41.4	38.7	9.6
*trnE(uuc)*	16,722	16,789	68				−	26.5	25.0	26.5	22.1

1) Dloop, Displacement loop; trnF (gaa), tRNA Phenylalanine; s-rRNA, short ribosomal RNA; trnV (uac), tRNA Valine; l-rRNA, length ribosomal RNA; trnL (uaa), tRNA Leucine; ND, NADH dehydrogenase; trnI (gau), tRNA Isoleucine; trnQ (uug), tRNA Glutamine; trnM (cau), tRNA Methionone; trnW (uca), tRNA Tryptophan; trnA (ugc), tRNA Alanine; trnN (guu), tRNA Asparagine; trnC (gca), tRNA Cystein; trnY (gua), tRNA Tyrosine; COX, Cytochrome c oxidase; trnS (uga), tRNA Serine; trnD (guc), tRNA Aspartic acid; trnK (uuu), tRNA Lysine; ATP, Adenosine triphosphate; trnG (uuc), tRNA Glycine; trnR (ucg), tRNA Arginine; trnH (gug), tRNA Histidine; CYTB, Cytochrome b; trnT (ugu), tRNA Threonine; trnP (ugg), tRNA Proline; trnE (uuc), tRNA Glutamic acid.

2) −, light strand; +, heavy strand.

**Table 3:** Length and nucleotide composition of mtDNA of *Gallus gallus*

No	Genbank ID	Breed	Length (bp)	Nucleotide composition (%)				Reference

				T	C	A	G	
1	-	Cemani chicken	16.789	23.7	32.5	30.3	13.5	This study
2	GU261688.1	Tengchongxue chicken	16.787	23.8	32.5	30.3	13.5	[19]
3	GU261690.1	Red Jungle Fowl	16.787	23.8	32.5	30.3	13.5	[19]
4	GU261676	Wuding chicken	16.778	23.8	32.5	30.3	13.5	[19]
5	GU261710	Nixi chicken	16.788	23.8	32.5	30.3	13.5	[19]
6	GU261680	Autochthonic chicken	16.786	23.7	32.5	30.3	13.5	[19]
7	GU261718	Lv’erwu chicken	16.786	23.7	32.5	30.3	13.5	[19]
8	GU261696	Jabouillei chicken	16.786	23.7	32.5	30.3	13.5	[19]
9	GU261675	Xuefeng chicken	16.785	23.7	32.5	30.3	13.5	[19]
10	GU261678	Gushi chicken	16.785	23.8	32.5	30.3	13.5	[19]
11	GU261719.1	Chigulu chicken	16.785	23.8	32.5	30.3	13.5	[19]
12	GU261714.1	Jiangbian chicken	16.785	23.8	32.5	30.3	13.5	[19]
13	KY039419	Kedu chicken	16.785	23.7	32.5	30.5	13.5	[20]
14	KF939304	Daweishan Mini chicken	16.785	23.7	32.5	30.3	13.5	[21]
15	OP718272	Yemili chicken	16.790	23.7	32.5	30.3	13.5	[22]
16	KX781318	Zhuxiang chicken	16.789	23.7	32.5	30.2	13.5	[23]
17	MT800327	White Layer chicken	16.789	23.7	32.6	30.3	13.5	[24]
18	AP003318	White Plymouth Rock chicken	16.785	23.7	32.5	30.3	13.5	[25]
19	AP003580.1	White Leghorn chicken	16.788	23.7	32.5	30.2	13.5	[25]
20	MN013407	Partridge shank chicken	16.788	23.7	32.5	30.2	13.5	[26]
21	KF954727	Huang Lang chicken	16.786	23.7	32.5	30.2	13.5	[27]
22	KM433666	Cenxi classical three-buff chicken	16.786	23.8	32.5	30.3	13.5	[28]
23	KM886937	Dong An Yellow chicken	16.786	23.7	32.5	30.3	13.5	[29]
24	KP269069	Nandan chicken	16.786	23.7	32.5	30.3	13.5	[30]
25	KP742951	Rugao Yellow chicken	16.786	23.8	32.5	30.3	13.5	[31]
26	MT773643.1	Huaibei Native chicken	16.786	23.7	32.5	30.3	13.5	[32]
27	MT177340.1	Gaojiao chicken	16.786	23.7	32.5	30.3	13.5	[33]
28	MN989997	Wannan Yellow chicken	16.786	23.7	32.5	30.2	13.5	[34]
29	MZ561474	Huaibei Partridge chicken	16.786	23.7	32.5	30.2	13.5	[35]
30	OP740915	Lac Son chicken	16.786	23.7	32.5	30.3	13.5	[36]
31	OP740917	Kien chicken	16.786	23.7	32.5	30.3	13.5	[37]
32	KM886936	Dong An Black chicken	16.785	23.8	32.5	30.3	13.5	[38]
33	KP244335	Hengshan Yellow chicken	16.785	23.7	32.5	30.3	13.5	[39]
34	OQ629493	Brahma chicken	16.784	23.8	32.4	30.3	13.5	[40]
34	KY039396.1	Manticao chicken	16.785	23.7	32.5	30.3	13.5	[20]
35	KY039385.1	Hawaiian chicken	16.785	23.7	32.5	30.2	13.5	[20]

**Table 4:** Polymorphic site of Dloop and protein-coding genes of Cemani chicken mtDNA aligned to references from genbank

Genes	Variable site		Parsimony			Singleton		
		
	n	(%)	n	(%)	Sites	n	(%)	Sites
*Dloop*	45	3.65	26	57.7	(199T>C); (212 G>A); (217T>C); (242G>A); (243C>T); (246C>T); (256C>T); (261T>C); (281G>A); (296C>A); (302C>T); (306C>T); (310T>C); (315C>T); (317A>C); (322T>C); (342A>G); (362C>T); (363C>T); 367T>C); (391C>A>T); (399G>A); (446C>T); (686G>A); (792G>A); (859Indel/C)	19	42.3	(167T>C); (219C>T); (225C>T); (234C>T); (236T>C); (250C>T); (254T>C); (265C>T); (291A>G); (341T>C); (347A>G); (355T>C); (649G>C); (890T>C); (309T>C); (1060A>G); (1080A>T); (860Indel/C); (988T/Indel)
*ND1*	9	0.92	3	33.3	(236C>T); (531G>A); (717G>A)	6	66.7	(28C>T); (174C>T); (327A>G); (340G>A); (396C>T); (774A>G)
*ND2*	7	0.67	5	71.4	(454A>T); (478G>T); (586T>C); (714A>G); (990G>A)	2	28.6	(292T>C); (401T>C)
*ND3*	5	1.42	2	40	(194T>C); (223C>T)	3	60	(249C>A); (280C>T); (328A>G)(MARKER!!!)
*ND4*	21	1.52	6	28.57	(9G>A); (198G>A); (478C>T); (609C>T); (969T>C); (1194C>T)	15	71.42	(78C>T); (161T>C); (189T>C); (200T>C); (484G>A); (513T>C); (640C>T); (934G>A); (1037G>A); (1078T>C); (1084G>A); (1087A>C); (1149T>C); (1195G>A); (1369A>G)
*ND4L*	1	0.33	1	100	(183C>T)	-	-	-
*ND5*	6	0.44	3	50	(24T>C); (339A>G); (854T>C)	3	50	(161C>A); (489C>G); (1102T>C)
*ND6*	10	1.91	5	50	(64T>C); (70C>T); (146G>A); (273T>C); (403G>A)	5	50	(76T>C); (163G>A); (178C>T); (363C>T); (367 G>A)
*COX1*	17	1.09	10	58.8	(114 T>C); (156 T>C); (255A>G); (381G>A); (564C>T); (826C>T); (1066G>A); (1237G>A); (1430T>C); (1543A>G)	7	41.2	(64A>C); (361G>A); (618G>A); (735C>T); (814C>T); (1178 G>C); (1410 T>C)
*COX2*	6	0.88	3	50	(134T>C); (279T>C); (303T>C)	3	50	(19C>T); (444C>T); (300A>G)
*COX3*	7	0.89	4	57.14	(50A>G); (339G>A); (516C>T); (738C>T)	3	42.86	(184G>A); (369C>T); (470A>C)
*ATP6*	8	1.17	4	50	(192G>A); (294A>G); (354A>G); (558A>G)	4	50	(66A>G); (308T>C); (600A>G); (666C>T)
*ATP8*	-	-	-	-	-	-	-	-
*CYTB*	15	1.31	9	60	(115G>A); (309A>G); (330C>T); (429G>A); (489G>A); (498A>G); (507C>T); (543C>T); (804T>C)	6	40	(199G>A); (390C>G); (933T>C); (927C>T); (1074T>C); (1090C>A)

**Table 5:** Amino acid alteration on protein-coding genes of Cemani chicken mtDNA aligned to references from genbank

PCGs	Mutation (n)		Codon	Amino acid alteration

	(n)	(%)		
*ND1*	2	0.61	(79ACT>ATT) (114GCC>ACC)	Threonine>Isoleucine Alanine>Threonine
*ND2*	4	1.16	(134ATA>ACA) (152ACC>TCC) (160GCC>TCC) (160GCC>TCC) (196TAT>CAT)	Methionine>Threonine Threonine>Serine Alanine>Serine Tyrosine>Histidine
*ND3*	5	4.27	(65TTT>TCT) (75CCC>TCC) (83TGC>TGA) (94CAC>TAC) (110AGG>GGG)	Phenylalanine>Serine Proline>Serine Cysteine>Tryptophan Histidine>Tyrosine *> Glycine
*ND4*	9	1.96	(54CTC>CCC) (67GTA>GCA) (162GTC>ATC) (312GCA>ACA) (346CGA>CCA) (362GCC>ACC) (363AAC>CAC) (399GCA>ACA) (457ACC>GCC)	Leucine>Proline Valine>Alanine Valine>Isoleucine Alanine>Threonine Arginine>Proline Alanine>Threonine Asparagine>Histidine Alanine>Threonine Threonine>Alanine
*ND4L*	-	-	-	
*ND5*	3	0.67	(54CCC>CAC) (285ATC>ACC) (368TCC>CCC)	Proline>Histidine Isoleucine>Threonine Serine>Proline
*ND6*	8	4.62	(22TAA>CAA) (24CAA>TAA) (26TCA>CCA) (49AGC>AAC) (55GCC>ACC) (60CAC>TAC) (123AGA>GGA) (135ACA>GCA)	*>Glutamine Glutamine>* Serine>Proline Serine>Asparagine Alanine>Threonine Histidine>Tyrosine Glycine>* Alanine> Threonine
*COX1*	7	1.35	(22ATT>CTT) (121GTC>ACT) (272CAT>TAT) (356GGG>AGG) (393AGG>ACG) (413GGC>AGC) (477CCT>CTT) (515ACA>GCA)	Isoleucine>Leucine Alanine>Threonine Histidine>Tyrosine Glycine>* *>Threonine Glycine>Serine Leucine> Proline Threonine>Alanine
*COX2*	1	0.44	(45ATA>ACA)	Methionine>Threonine
*COX3*	2	0.76	(62GTC>ATC) (157AAA>ACA)	Valine>Isoleucine Lysine>Threonine
*ATP6*	1	0.44	(103ATG>ACG)	Methionine>Threonine
*ATP8*	-	-	-	-
*CYTB*	3	0.79	(39GCA>ACA) (67GTA>ATA) (364CTT>ATT)	Alanine> Threonine Valine>Methionine Leucine>Isoleucine

PCGs, protein-coding genes.
